# Visualizing Single-Cell RNA-seq Data with Semisupervised Principal Component Analysis

**DOI:** 10.3390/ijms21165797

**Published:** 2020-08-12

**Authors:** Zhenqiu Liu

**Affiliations:** Department of Public Health Sciences, Penn State College of Medicine, Hershey, PA 17033, USA; zliu3@phs.psu.edu

**Keywords:** scRNA-seq visualization, semisupervised principal component analysis, dimension reduction, cluster visualization, nonlinear visualization

## Abstract

Single-cell RNA-seq (scRNA-seq) is a powerful tool for analyzing heterogeneous and functionally diverse cell population. Visualizing scRNA-seq data can help us effectively extract meaningful biological information and identify novel cell subtypes. Currently, the most popular methods for scRNA-seq visualization are principal component analysis (PCA) and t-distributed stochastic neighbor embedding (t-SNE). While PCA is an unsupervised dimension reduction technique, t-SNE incorporates cluster information into pairwise probability, and then maximizes the Kullback–Leibler divergence. Uniform Manifold Approximation and Projection (UMAP) is another recently developed visualization method similar to t-SNE. However, one limitation with UMAP and t-SNE is that they can only capture the local structure of the data, the global structure of the data is not faithfully preserved. In this manuscript, we propose a semisupervised principal component analysis (ssPCA) approach for scRNA-seq visualization. The proposed approach incorporates cluster-labels into dimension reduction and discovers principal components that maximize both data variance and cluster dependence. ssPCA must have cluster-labels as its input. Therefore, it is most useful for visualizing clusters from a scRNA-seq clustering software. Our experiments with simulation and real scRNA-seq data demonstrate that ssPCA is able to preserve both local and global structures of the data, and uncover the transition and progressions in the data, if they exist. In addition, ssPCA is convex and has a global optimal solution. It is also robust and computationally efficient, making it viable for scRNA-seq cluster visualization.

## 1. Introduction

Single-cell RNA sequencing (scRNA-seq) technology enables the measurement of cell-to-cell expression variability of thousands to hundreds of thousands of genes simultaneously, and provides a powerful approach for the quantitative characterization of cell types based on high-throughput transcriptome profiles. A full characterization of the transcriptional landscape of individual cells has enormous potential for both biological and clinical applications. However, characterization and identification of cell types require robust and efficient computational methods. Particularly, visualization is crucial for humans interactively processing and interpreting the heterogeneous and high-dimensional scRNA-seq data, because humans rely on their astonishing cognitive abilities to detect visual structures, such as clusters and outliers. Hence, high-dimensional data must be projected (embedded) into a 2D or 3D space with dimension reduction (DR) techniques for visualization.

Among different methods proposed for scRNA-seq visualization, the two popular ones are principal component analysis (PCA) and t-distributed Stochastic Neighbor Embedding (t-SNE). PCA projects the high-dimensional scRNA-seq data into the linearly orthogonal low-dimensional vector space thorough variance maximization. Due to its efficiency and conceptual simplicity, PCA has been widely applied to scRNA-seq data dimension reduction and visualization [1,2,3,4,5,6]. Several methods utilized PCA as a data preprocessing step for scRNA-seq clustering. For instance, principal components from distance matrix were used for consensus clustering in SC3 [3], and the low-dimensional orthogonal representations through iterative PCA were implemented in pcaReduce [4]. However, PCA is unsupervised and linear. It discovers the directions along the maximum variation, ignores the information of cell clusters, and fails to detect the nonlinear relationship among cells. But it is critical to project scRNA-seq data onto the directions correlated with cell subtypes for clustering visualization.

On the other hand, t-distributed Stochastic Neighbor Embedding (t-SNE) is the most commonly used nonlinear dimension reduction method for cell subtype visualization. t-SNE transforms cell similarities into probability, and incorporates the information of cell clusters into visualization through redefining the probability. It determines the spatial cell maps in low dimension through minimizing the Kullback–Leibler divergence [7]. t-SNE recently became a standard tool for dimension reduction and scRNA-seq visualization, and has been implemented in many software tools [8,9,10,11,12,13]. Nonetheless, the cost function of t-SNE with Kullback–Leibler divergence minimization is not convex, so that the solution may stick to a local minimum. The free parameters of t-SNE also need to be tuned. Most importantly, t-SNE fails to preserve global data structure, indicating that the intercluster relations are meaningless. In addition, t-SNE is not computationally scalable for large problems.

Uniform Manifold Approximation and Projection (UMAP) is a new scRNA-seq visualization software [14,15]. Similar to t-SNE, UMAP constructs a high-dimensional graph representation of the data, then builds a low-dimensional graph that is as structurally similar as possible. Both UMAP and t-SNE are based on the k-nearest neighbor graph technique that only ensures the local connectivity of the manifold. It has been demonstrated that UMAP is computationally more efficient than t-SNE. However, the intercluster distances are still not meaningful due to the local neighbor graph approach used in UMAP.

In this paper, we propose a semisupervised principal component analysis (ssPCA) method for dimension reduction and visualization of scRNA-seq data. ssPCA is a generalization of PCA and it is nonlinear. It seeks to find principal components that maximize both data variance and cluster dependence, so that cluster (subtype) labels are integrated into scRNA-seq visualization seamlessly. While maximizing the total data variance preserves the global structure in the data, maximizing the cluster dependence captures the local data structure within each cluster. In addition, ssPCA can be solved in a closed-form and does not suffer from the high computational complexity of iterative optimization procedures. Therefore, it is computationally efficient.

## 2. Materials and Methods

### 2.1. The Methods

We propose a semisupervised principal component analysis (ssPCA) approach for the visualization of scRNA-seq data. The proposed approach is based on nonlinear kernel (or similarity) matrices and incorporates cluster labels into the visualization. This optimization problem is involved in two components, including the unsupervised maximization of total variance and the supervised maximization of cluster dependence with the Hilbert–Schmidt Independence Criterion (HSIC). Overall, the proposed approach is, therefore, a semisupervised learning problem. In addition, because cluster labels are required for ssPCA visualization, ssPCA is most useful for the visualization of clusters detected from other scRNA-seq clustering tools. Note that we can easily construct a kernel matrix for ssPCA if there is no similarity matrix available from other clustering software (which is rare).

Given a n×p scRNA-seq data matrix *X* with *n* cells and *p* genes, and a n×1 cell cluster vector y, we aim to project the scRNA-seq data onto low-dimensional orthogonal space for cluster visualization. The idea to incorporate the clustering information into principal component analysis based on HSIC. HSIC is a standard method for measuring the dependence between two sets of random variables [16]. With a scRNA-seq data *X* and cell subtype (cluster) vector y, two kernels $K_{X}\in R^{n\times n}$ and $K_{Y}\in R^{n\times n}$ are built from *X* and y, respectively. Then, the empirical estimate of HSIC is

$$HSIC\left(X,y\right)=\frac{1}{{\left(n-1\right)}^{2}}tr\left(K_{X}HK_{Y}H\right),\;\;\;\mathrm{where}\;\;\;H=I-\frac{1}{n}1_{n}1_{n}^{T}\in R^{n\times n},$$

where *H* is the centering matrix and $1_{n}$ is a n×1 vector of all 1s. Maximizing HSIC will maximize the dependence between cell expression and cluster labels. Supervised PCA with HSIC and related optimization has been explored in different studies [17,18].

Our proposed ssPCA method for cluster visualization aims not only to maximize HSIC, but also to preserve the data variance. Kernel measures the inner-product in a new feature space. In general, a linear or nonlinear function Φ(X) is used to map the data *X* onto a new space, then a kernel is defined as $K_{X}=\Phi \left(X\right)\Phi {\left(X\right)}^{T}$. With the kernel trick, we can define the kernel directly without knowing the exact form of Φ. Given the expressions of two cells $x_{i}$ and $x_{j}$, common kernels include linear ($K\left(x_{i},x_{j}\right)=x_{i}^{T}x_{j}$), polynomial ($K\left(x_{i},x_{j}\right)={\left(cx_{i}^{T}x_{j}+1\right)}^{d}$), and radial basis functions ($K\left(x_{i},x_{j}\right)=\exp(-||x_{i}-x_{j}{\left|\right|}^{2}/\sigma^{2})$). Kernel method has been used for scRNA-seq clustering [19]. The similarity matrix generated from any scRNA-seq clustering software can be used as a kernel ($K_{X}$) for cluster visualization.

The kernel for cluster (subtype) labels y is defined as follows. Suppose there are *c* cell clusters in y, we first recode y into a binary matrix $Y\in R^{n\times c}$ with the one-hot coding scheme—i.e., $Y\in R^{n\times c}$, where $y_{ij}=1$ if the cell *i* belongs to cluster *j*, and is 0 otherwise. Then, we define the kernel $K_{Y}$ as

$$K_{Y}=YY^{T}.$$

With the kernel matrices $K_{X}$ and $K_{Y}$ available, we project the kernel $K_{X}$ onto a low-dimensional $Z\in R^{n\times k}$ (where k=2 or 3) for cluster visualization.

$$Z=K_{X}W,\;\;\;\mathrm{where}\;\;\;W\in R^{n\times k}\mathrm{is}\mathrm{the}\mathrm{projection}\mathrm{coefficient}\mathrm{matrix}.$$

The linear kernel in the low-dimensional space is $K_{Z}=ZZ^{T}=K_{X}WW^{T}K_{X}$. Therefore, after dropping the scaling factor, we have the supervised HSIC maximization in the projected low-dimensional space as

$$tr\left(K_{Z}HK_{Y}H\right)=tr\left(K_{X}WW^{T}K_{X}HK_{Y}H\right)=tr\left(W^{T}K_{X}HK_{Y}HK_{X}W\right).$$

The second term is the unsupervised total variance maximization in the projected space. The variance covariance matrix $S_{Z}$

$$S_{Z}=cov\left(Z\right)=Z^{T}HZ=W^{T}K_{X}HK_{X}W,$$

where $H=I-\frac{1}{n}1_{n}1_{n}^{T}$, which is the same as we defined previously. Put the two terms for HSIC and total variance ($tr(S_{Z})$) together and add an orthogonal constraint, $W^{T}W=I$; we optimize the following semisupervised PCA problem:

$$\begin{array}{cc} \underset{W}{arg max} & \;\{\left(1-\lambda \right)tr\left(W^{T}K_{X}HK_{X}W\right)+\lambda tr\left(W^{T}K_{X}HK_{Y}HK_{X}W\right)\}\\ s.t.:\;\; & W^{T}W=I, \end{array}$$

where 0≤λ≤1 is a trade-off hyperparameter. When λ=0, the problem becomes the traditional unsupervised kernel PCA. When λ=1, the problem is the supervised PCA. With the Lagrangian multiplier method, the solution for *W* is the eigenvectors of $\left(1-\lambda \right)K_{X}HK_{X}+\lambda K_{X}HK_{Y}HK_{X}$ corresponding to the *k* largest eigenvalues. The projection $Z=K_{X}W$ will be used for visualization.

ssPCA is most suitable for visualizing cell clusters detected from other scRNA-seq software, since cluster labels are required for ssPCA visualization. Both the cluster labels and the similarity (distance) matrix used for cell clustering are usually available. In such a case, the similarity matrix is treated as the kernel ($K_{X}$) for *X*, $K_{Y}=YY^{T}$ is constructed from cluster labels *Y*, and the low-dimensional projection will be discovered and visualized with ssPCA. If only cluster labels are available (which is rare), we can construct our own kernel $K_{X}$ for visualization. The ssPCA algorithm (Algorithm 1) for cluster visualization of scRNA-seq data is as follows:
**Algorithm 1:**The ssPCA algorithm Given the cluster labels y, scRNA-seq data *X*, similarity (kernel) matrix $K_{X}$ and hyperparameter λ:  1. Recode y into a binary matrix *Y*, calculate $K_{Y}=YY^{T}$, and compute       $K_{X}$ from *X* if $K_{X}$ is not available.   2. Find *W*, the eigenvectors of $\left(1-\lambda \right)K_{X}HK_{X}+\lambda K_{X}HK_{Y}HK_{X}$ corresponding to       the *k* largest eigenvalues.   3. Project to low-dimension with $Z=K_{X}W$ for cluster visualization. 


**The hyperparameter λ:** We demonstrate that the visualization graphs are very similar across different λs with the simulation data. Thus, the proposed method is quite robust with λ. We set λ=0.75 in all computational experiments with real data for comparison.

### 2.2. The scRNA-seq Datasets

Four scRNA-seq datasets are used for evaluation. They span a wide range of cell types with known numbers of subpopulations, representing a broad spectrum of single-cell data. The first dataset consists of embryonic stem cells under different cell cycle stages [2], which includes 8989 genes, 182 cells, and 3 known cell subtypes. The second dataset contains pluripotent cells under different environment conditions [20], which has 10,685 genes, 704 cells, and 3 cell subtypes. The third one is composed of eleven cell populations including neural cells and blood cells [21], which contains 14,805 genes, 249 cells, and 11 cell subtypes. The fourth dataset consists of neuronal cells with sensory subtypes [5], which includes 17,772 genes, 622 cells, and 4 cell subtypes. Cell subtypes in each data are known in advance, providing nice data sources for performance evaluation.

## 3. Results

### 3.1. Simulation Data

**Figure 1:**
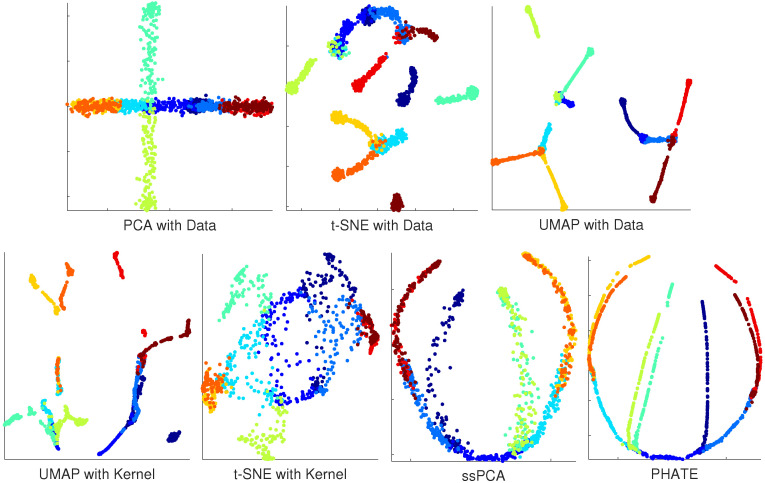
Visualization results with artificial tree data. **Top panel from left to right:** principal component analysis (PCA) with Data; t-distributed stochastic neighbor embedding (t-SNE) with Data, and Uniform Manifold Approximation and Projection (UMAP) with Data. **Bottom panel from left to right:** UMAP with Kernel; t-SNE with Kernel; ssPCA with the same Kernel; and PHATE (the ground-truth).

It is challenging to compare the performance of different visualization software. One reliable approach is to perform simulations with ground-truth available. Our simulation is based on the artificial tree data from PHATE (Potential of Heat-diffusion for Affinity-based Transition Embedding) [22]. PHATE was recently developed with a novel informational distance developed from diffusion processes, and was an efficient tool for visualizing continuous progression and trajectories. The artificial tree included 10 branches, and data for each branch was uniformly sampled in 60 dimensions. The sample size of the tree data was 1440 with branch information available, providing a nice source for ssPCA assessment and software comparisons. The goal for this visualization is not only to detect the clusters (local data structure), but also to recover the branching trajectories of the simulated tree correctly. Note that PHATE is also a visualization software. To evaluate the true power of ssPCA, compared to PCA, t-SNE, and UMAP, we did not use the final similarity matrix learned from PHATE, but constructed a simple Gaussian kernel matrix from the data, and the 10 branches were regarded as the cluster labels. The same kernel was used to evaluate the performance of UMAP, t-SNE, and ssPCA, respectively. With the data dimensions of 60, no PCA is carried out for t-SNE and UMAP with original tree data. However, PCA is used for dimension reduction before we perform UMAP and t-SNE with kernel. The number of PCs used for UMAP and t-SNE is set to 30 in this simulation. The results with different methods are reported in Figure 1.

**Figure A1:**
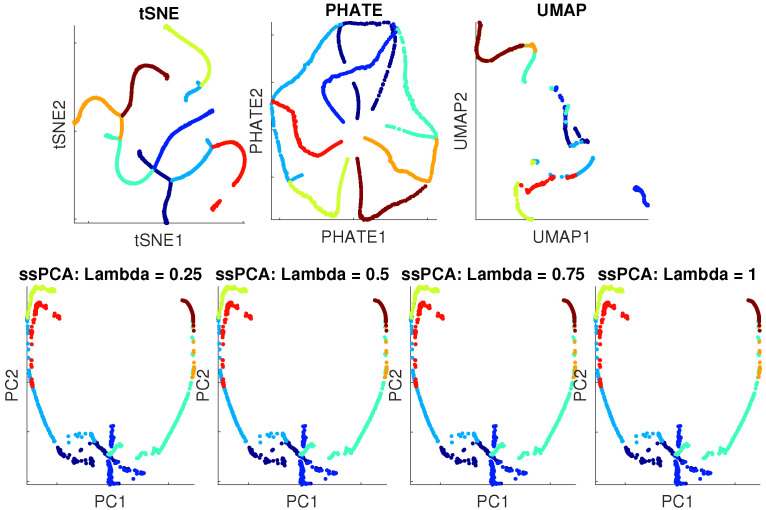
Visualization results with artificial tree data with the noise of σ=3. **Up-left:** t-SNE; **Up-middle:** PHATE; **Up-right:** UMAP; **Bottom panels from left to right:** ssPCA with λ=0.25, 0.5, 0.75, and 1, respectively.

**Figure A2:**
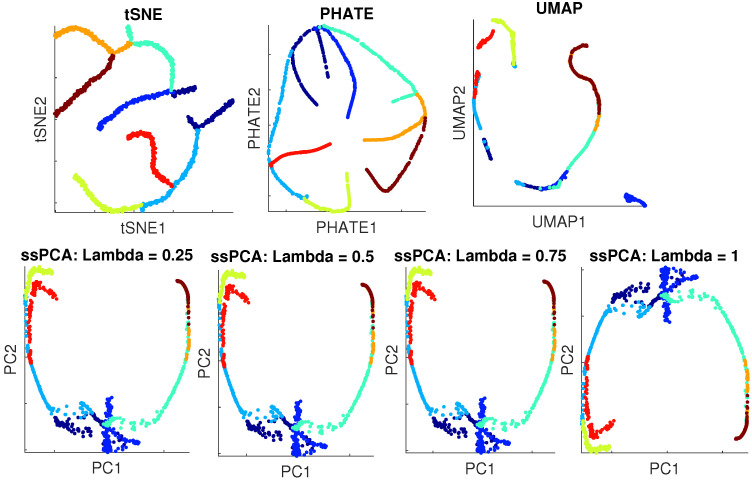
Visualization results with artificial tree data with the noise of σ=6. **Up-left:** t-SNE; **Up-middle:** PHATE; **Up-right:** UMAP; **Bottom panels from left to right:** ssPCA with λ=0.25, 0.5, 0.75, and 1, respectively.

**Figure A3:**
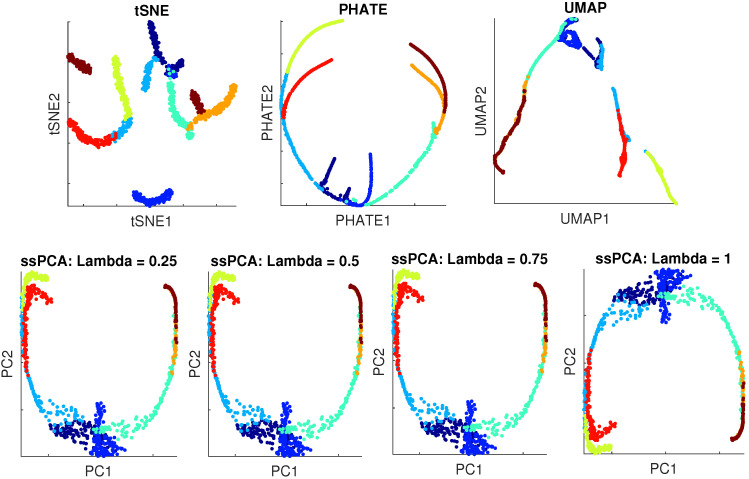
Visualization results with artificial tree data with the noise of σ=12. **Up-left:** t-SNE; **Up-middle:** PHATE; **Up-right:** UMAP; **Bottom panels from left to right:** ssPCA with λ=0.25, 0.5, 0.75, and 1, respectively.

Figure 1 demonstrates that neither PCA nor t-SNE recover the tree structure of the data correctly. PCA leads to artificially overlapping, while t-SNE with data shatters the tree structure into discrete clusters, and we cannot recognize the tree structure from t-SNE with kernel, indicating that t-SNE does not preserve the global data structure. UMAP with both data and kernel seems to be better at preserving the global tree structure, a few tree branches are connected together. However, tree branches in UMAP are also shattered into discrete pieces. The global tree structure is not completely recovered and the intercluster distances are meaningless with UMAP. PHATE performs the best and represents the ground-truth with this artificial tree data. It correctly visualizes the global and local tree structures. Although ssPCA is mainly designed for visualizing cluster structures, it performs second-best and correctly visualizes the global tree structures. The intercluster distances in the artificial tree data are correctly preserved with ssPCA. ssPCA also discovers most of the clusters (branches) correctly. It only fails to distinguish a couple of tree branches that are close to each other because of the noises. PHATE performs better than ssPCA with this specific dataset, but not without the costs. There are more than 5 hyperparameters in PHATE, and different parameter settings may lead to quite different visualization results. Particularly, PHATE is sensitive to the choice of the noise parameter σ. The visualizations of PHATE are quite different with different noises σs, although the true structure of the artificial tree is the same. Additional simulations are carried out with the parameters set to (i) the number of dimensions of 5000; (ii) number of samples of 1200; (iii) the number of branches of 8; and (iv) the different noise levels of σ=3, 6, and 12, respectively. PCA is performed for dimension reduction before we carry out UMAP and t-SNE. The number of PCs is also set to 30 with more than 70% of explained variance. The visualization results are reported in Appendix A Figure A1, Figure A2 and Figure A3. PHATE leads to overfitting the tree-structure when the noise in the data is relatively low (σ=3 and σ=6), but performs better with higher noises (σ=12). ssPCA on the other hand, is robust with different choices of the hyperparameter (λ=0.25, 0.5, 0.75, and 1) and different noises (σ=3, 6, and 12), as presented in the top-right and bottom panels of Figure A1, Figure A2 and Figure A3. ssPCA also performs better than UMAP. In addition, ssPCA is computationally more efficient than PHATE, t-SNE, and OMAP. These three software utilize a gradient-decent approach to find the optimal solution, which is relatively time-consuming. The computational time for PCA, ssPCA, t-SNE, UMAP, and PHATE are 0.018, 1.96, 41.88, 8.05, and 13.24 s, respectively, with the artificial tree data running on an Intel core i7 laptop with 12-GB memory. In conclusion, ssPCA preserves both global and local structures of the data. When transition and progressions exist in the branches of artificial tree, ssPCA captures the branching trajectories well.

### 3.2. Real Data

Our computational evaluations with four real datasets are based on the cell similarity matrix and clustering labels from three popular scRNA-seq software including SIMLR [19], SoptSC [23], and sinNLRR [24]. t-SNE was implemented in their original packages for visualization. As PHATE is not solely designed for cluster visualization, we only compare the performances of ssPCA with t-SNE and UMAP using the real datasets. Note that PCA is carried out for dimension reduction before we perform UMAP and t-SNE. The number of PCs is set to 20, with more than 70% of explained variance for all 4 datasets.

**Figure 2:**
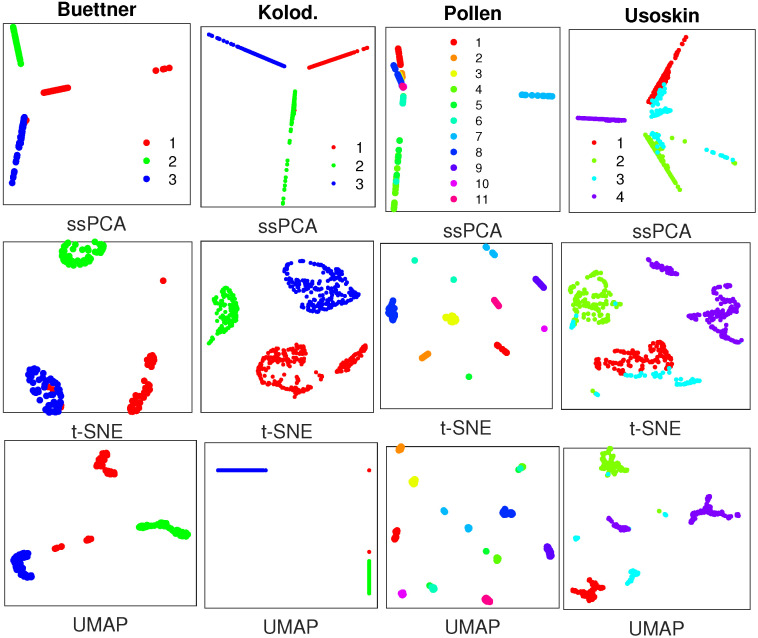
Visualization results with similarity matrices from SIMLR with 4 real single-cell RNA sequencing (scRNA-seq) datasets, where plots in the top row are generated by semisupervised principal component analysis (ssPCA) with λ=0.75, and plots in the middle row and bottom row are produced with t-distributed stochastic neighbor embedding (t-SNE) and UMAP, respectively. Datasets to draw the the subplots from left to right: Buettner [2]; Kolodziejczyk [20]; Pollen [21]; Usoskin [5].

**SIMLR** [19] is denoted as single-cell interpretation via multikernel enhanced similarity learning. It learns the cell-to-cell similarities through efficient multikernel optimization. The similarity matrices from SIMLR for the 4 real single-cell datasets are visualized with ssPCA, t-SNE, and UMAP, respectively. The perplexity value for t-SNE and the number of nearest neighbors for UMAP are set to 30. We actually try several perplexity values (10, 20, 30, 40) and choose the one with a better visualization. The clusters with different scRNA-seq data are visualized in Figure 2.

While ssPCA, t-SNE, and UMAP can separate the clusters in different datasets with the similarity matrices from SIMLR, ssPCA performs better in recovering the global cluster structures relative to one another. For instance, with the Buettner scRNA-seq data, ssPCA demonstrates that cluster 2 (green) is close to cluster 1 (red), and far away from cluster 3 (blue), while the relative locations (distances) of the three clusters with t-SNE and UMAP might not mean anything. In addition, with the Pollen data, t-SNE and UMAP display all clusters uniformly on the plane, while ssPCA shows that cluster 7 (light-blue) is far away from the rest clusters. Finally, with the Usoskin data, cluster 3 (light blue) is displayed as several pieces with t-SNE and UMAP, but it is visualized as one entity and adjacent to cluster 1 (red) and cluster 2 (green) with ssPCA. This is reasonable. As demonstrated with the simulation data, ssPCA preserves both global and local data structures with principal component projection, while t-SNE and UMAP only capture the local data structure, and the global structure is not fully preserved. The main reason is that both t-SNE and UMAP are based on the nearest neighbor graph technique which only optimizes the data points close to each other.

**Figure 3:**
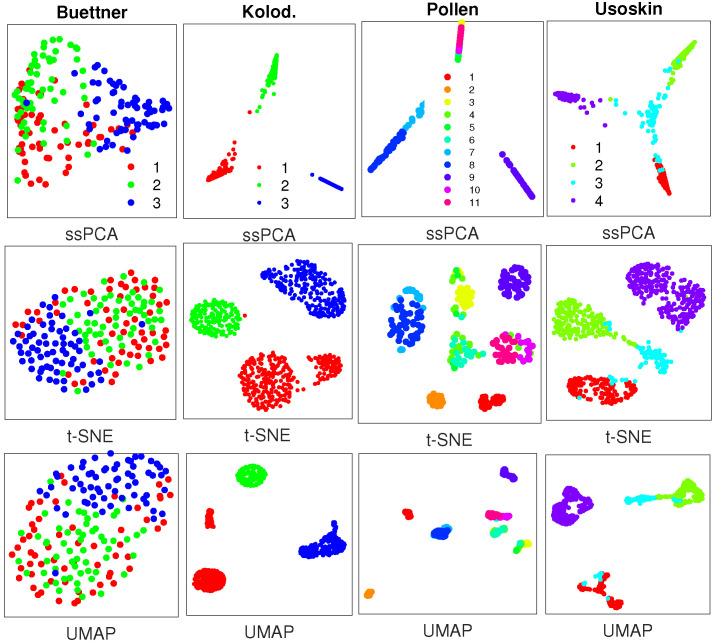
Visualization results with similarity matrices from SoptSC with 4 real scRNA-seq datasets, where plots in the top row are generated by ssPCA with λ=0.75, and plots in the middle and bottom rows are produced with t-SNE and UMAP, respectively. Datasets to draw the the subplots from left to right: Buettner [2]; Kolodziejczyk [20]; Pollen [21]; Usoskin [5].

**SoptSC** [23] is a recently developed software that learns cell–cell similarities through locality-preserving low-rank representation. The similarity matrices from the same 4 scRNA-seq datasets are visualized with ssPCA, t-SNE, and UMAP, respectively, as shown in Figure 3.

**Figure A4:**
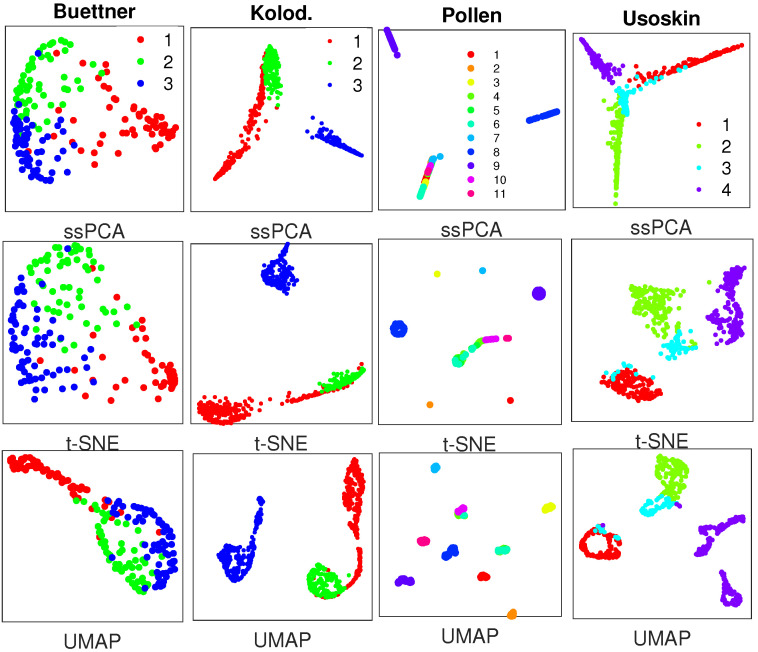
Visualization results from sinNLRR with 4 real scRNA-seq datasets, where plots in the top row are generated by ssPCA with λ=0.75, and plots in the middle and bottom rows are produced with t-SNE and UMAP, respectively. Datasets to draw the the subplots from left to right: Buettner [2]; Kolodziejczyk [20]; Pollen [21]; Usoskin [5].

Figure 3 demonstrates that ssPCA, t-SNE, and UMAP can separate the clusters in 3 out of 4 datasets including Kolodziejczyk, Pollen, and Usoskin with the similarity matrices from SoptSC. However, for the Buettner scRNA-seq data, t-SNE and UMAP fail to distinguish cluster 1 (red) from cluster 2 (green) and cluster 3 (blue), while ssPCA is able to recover these clusters as separate entities, although there are some overlaps among them. With the other 3 datasets, ssPCA provides additional information about the clusters relative to one another. For instance, with the Usoskin dataset, cluster 3 (light blue) is located at the center, and links clusters 1, 2, and 4 together with ssPCA, while it is hard to identify such related information of the clusters with t-SNE and UMAP, because the intercluster distances with t-SNE and UMAP are meaningless. Similar visualization results with sinNLRR [24] are reported in Figure A4 of the Appendix A. SoptSC and sinNLRR are based on the same idea of locality-preserving low-rank representation. The only difference is that F-norm is used in SoptSC, while $L_{21}$ norm is used in sinNLRR. Thus, the similarity matrices generated by SoptSC and sinNLRR with the 4 scRNA-seq data are comparable, leading to similar visualization results.

## 4. Discussion

Visualization of the high-dimensional RNA-seq data is critical for detecting cell subpopulations and revealing biological insights. To date, there are only a few tools including PCA, t-SNE, UMAP, and PHATE available for dimension-reduction and scRNA-seq data visualization. ssPCA provides another viable tool for scRNA-seq visualization and has its advantages. More specifically, PCA projects high-dimensional data into a low-dimensional space through eigenvalue decomposition. PCA is a linear projection method, and it mainly captures the global structure of the data, as demonstrated in Figure 1 of the artificial tree visualization. However, scRNA-seq data are usually not linear, and the nonlinear structure in scRNA-seq data cannot be detected by PCA. ssPCA, on the other hand, is a nonlinear kernel extension of PCA. It reduces the nonlinear noises and projects the scRNA-seq data onto a low-dimensional manifold.

t-SNE and UMAP are the two popular local graph algorithms for scRNA-seq data visualization. While t-SNE is the most popular tool currently used in the literature, UMAP produces somewhat similar output with increased speed. However, one key disadvantage with t-SNE and UMAP is that they only preserve the local neighborhood structure in the data. The global structures are not correctly visualized. As demonstrated in Figure 1 and Appendix A Figure A1, Figure A2 and Figure A3, both t-SNE and UMAP tends to shatter the continuous structures into discrete clusters, and the relative location of clusters in t-SNE and UMAP generally has no meaning. ssPCA, on the other hand, integrates local cluster dependence with global principal components. It is able to maintain both global and local structures, as demonstrated in Figure 1 and Figure A1, Figure A2 and Figure A3. Moreover, in practice, both t-SNE and UMAP utilize PCA as a dimension reduction prior, because of the large number of genes in scRNA-sq data. Although UMAP can handle the high-dimensional data efficiently, PCA for dimension reduction is still necessary, due to the curse of dimensionality. The distance between cells in high-dimension tends to be very similar, leading to deteriorating performance in cluster visualization. On the contrary, ssPCA finds the principal components in one-step, which is computationally more efficient.

PHATE [22] is a recently developed software for dimension reduction and visualization. It visualizes the simulated tree structure better when the noise in the artificial tree data is high, but tends to overfit the data and leads to a too-complex visualization when the noise is relatively low, as demonstrated in Figure 1 and Appendix A Figure A1, Figure A2 and Figure A3. Furthermore, there are too many parameters in PHATE, and different parametric settings may lead to different visualizations. On the contrary, ssPCA only has one hyperparameter λ, and it is robust with different values of λ over different noise levels, as demonstrated in Figure A1, Figure A2 and Figure A3.

The only hyperparameter λ (0≤λ≤1) measures the trade-off between the total variance of the data and cluster dependence. When λ=0, ssPCA becomes a standard kernel PCA that maximizes the total variance. On the other hand, when λ=1, the projection is solely based on maximizing cluster dependence of the data. As ssPCA is robust with different values of λ in the simulation, we recommend that you pick a λ value between 0.25–0.75 in practice. Since ssPCA is computational efficient, you may run ssPCA multiple times with different values of hyperparameter λ, and get a better sense of how the projection is affected by λ.

One interesting finding with the Pollen data is that ssPCA identifies 3 distinct clusters with different kernel (similarity) matrices from different software packages, while 11 clusters were discovered in their original study. Whether this discrepancy is from the novel finding with ssPCA or from the limitation of ssPCA is not known. More investigations are required in the near future. Finally, although the same 4 scRNA-seq datasets are used for Figure 2 and Figure 3, the visualizations are not exactly the same with different similarity matrices from different software. Thus, the choice of similarity (kernel) matrices is also crucial for scRNA-seq visualization.

## 5. Conclusions

We propose a semisupervised principal component method (ssPCA) with HSIC maximization for cell subtype visualization. ssPCA optimizes both local cluster dependence and global principal component projection. It has an analytical solution, and is robust with respect to different values of hyperparameter λ and different noise levels in the data. Thus, ssPCA has its advantages over PCA, t-SNE, UMAP, and PHATE. The key advantages with ssPCA are that it preserves both local and global structures in the data faithfully, and the principal component projection with ssPCA is more interpretable than that from t-SNE and UMAP. However, it is important to remember that no visualization technique is perfect, and ssPCA is no exception. Unlike t-SNE and UMAP, in which cluster labels are only optional, ssPCA must have cluster labels as its input, indicating that ssPCA can only be used when cluster information is available. However, through integration with a clustering software, ssPCA is still a powerful tool for scRNA-seq data visualization. It also provides an alternative to standard visualization methods such as t-SNE and UMAP.

## Abbreviations

The following abbreviations are used in this manuscript:

PCA	Principal Component Analysis
ssPCA	Semisupervised Principal Analysis
t-SNE	t-distributed Stochastic Neighbor Embedding
scRNA-seq	Single Cell RNA-sequencing
PHATE	Potential of Heat-diffusion for Affinity-based Transition Embedding
UMAP	Uniform Manifold Approximation and Projection

## Appendix A. Supplementary Figure A1, Figure A2, Figure A3 and Figure A4

We did more simulations with artificial tree structure visualization where the parameters were set to the following: (i) the number of dimensions of 5000; (ii) number of samples of 1200; (iii) the number of branches of 8; and (iv) the different noises of σ=3, 6, and 12, respectively.
